# Caregivers’ Mastery in Handling Gastrostomy at Home after Educational Intervention: Qualitative Descriptive Study

**DOI:** 10.3390/healthcare12212147

**Published:** 2024-10-29

**Authors:** Jeferson Moreira dos Santos, Larissa Chaves Pedreira, Roberta Pereira Góes, Maria Antônia Alves de Souza, Cristina Rosa Soares Lavareda Baixinho, Johis Ortega, Rosseirys Noelia De La Rosa, Anderson Reis Sousa, Valdenir Almeida da Silva, Ivana Santos Pinto, Jéssica Lane Pereira Santos, Letícia Chicharo Vivas, Lélia Mendes Sobrinho de Oliveira

**Affiliations:** 1 School of Nursing, Federal University of Bahia, Salvador 40110-907, Brazil; larissa.pedreira@uol.com.br (L.C.P.); robertapgoes@yahoo.com.br (R.P.G.); anderson.sousa@ufba.br (A.R.S.); valdenir.silva@ebserh.gov.br (V.A.d.S.); stos_ivana@yahoo.com.br (I.S.P.); jessicalane84@gmail.com (J.L.P.S.); leticiacv@ufba.br (L.C.V.); leliasobrinho79@gmail.com (L.M.S.d.O.); 2 Nursing Department, University of the State of Bahia, Guanambi 46360-000, Brazil; mariantoniabh@gmail.com; 3 Center for Research, Innovation and Development in Nursing, Lisbon School of Nursing, 1990-096 Lisboa, Portugal; crbaixinho@esel.pt; 4 School of Nursing and Health Studies, University of Miami, Coral Gables, FL 248153, USA; j.ortega10@miami.edu (J.O.); rosseirys.de.la.rosa@vanderbilt.edu (R.N.D.L.R.)

**Keywords:** gastrostomy, hospitalization, hospital to home transition, family caregiver

## Abstract

Background: Effective hospital discharge planning is crucial, particularly in educating caregivers on handling medical devices. This education helps manage the patient’s signs and symptoms, prevents post-discharge complications, and reduces early readmissions. This study aimed to understand aspects involved in the acquisition of mastery by home caregivers, in handling care of a patient who just underwent gastrostomy, after educational intervention during hospitalization and telemonitoring upon return home. Methods: Qualitative descriptive study. It followed 15 caregivers of people with percutaneous endoscopic gastrostomy. The intervention took place between November 2022 and July 2023 in the neuromusculoskeletal unit of a Brazilian university hospital. Results: The educational intervention had four stages. In stage 1, caregivers felt confused and uncertain about managing PEG. By stage 2, they expressed a desire to be capable of handling care, especially in the event of potential complications, and showed increased awareness and engagement. Stage 3 highlighted the effectiveness of hands-on training with feedback from professionals. In stage 4, during monitoring, several complications were noted, including granuloma formation in the stoma, tube obstruction, and accidental tube loss. However, caregivers demonstrated the ability to handle these situations, indicating the effectiveness of the training and telemonitoring interventions. Conclusions: Nursing professionals should consider various factors when training caregivers in a hospital setting, including providing adequate space, allocating sufficient time for educational interventions, offering both theoretical and practical demonstrations, ensuring effective communication, and taking into account the caregivers’ context, as they play a direct role in acquiring a safe and effective skill set.

## 1. Introduction

Percutaneous endoscopic gastrostomy (PEG) is a minimally invasive surgical procedure where a catheter is inserted directly into the stomach for feeding. It is widely recommended for use when long-term enteral nutrition is required, due to inadequate oral intake, which may occur due to neurological conditions affecting swallowing, such as stroke, motor neuron disease, and demyelinating diseases, among others [1,2].

The benefits of this device include ease of insertion, shorter hospitalization time, early start of nutritional support, better cost-effectiveness, and safety during feeding. However, complications can occur when specific guidance is not provided to the patient/caregiver for handling, such as adequate care and management, aiming at continuity at home [3].

The most common complications are mechanical and related to catheter obstruction, leakage related to stoma hypergranulation [4], and buried bumper syndrome, an uncommon adverse event, resulting from the tight positioning of the “pulley”, the external structure of the PEG tube, against the abdominal wall, causing swelling, erythema of the surrounding tissues, leaks around the tube, resistance and/or abdominal pain with the infusion of food [5]. Problems related to the gastrointestinal tract itself, such as nausea, vomiting, increased gastric residual volume, abdominal distension, diarrhea, and infection at the site of the surgical wound are also mentioned [6,7].

Furthermore, there may also be accidental loss of the tube, resulting in closure of the stoma and the need for a new approach. In the case of people dependent on the public health system, this demand causes and intensifies serious problems, such as when they need to return to the hospital for a new treatment. Stoma closure can occur between 24 and 72 h; however, this occlusion, in some cases, can occur between four and eight hours [8,9].

For this reason, health professionals must manage discharge, implementing transitional care from the moment the PEG is indicated, with support from the multidisciplinary team, with the nurse as the protagonist. In this care, assertive guidance and health education for affected people and/or their caregivers are necessary, through training in handling the tube, recognition and management of complications that can be referred and/or handled by the caregiver, skin care, and oral hygiene (regardless of whether or not the oral diet is maintained), as many people may be bedridden and need continued care.

Such actions are essential, as caregivers of people using PEG have problems due to the lack of training post discharge and express difficulties in dealing with onerous responsibilities related to food and stoma care at home [6,10].

Therefore, preparing these people for continuity of care is important, especially in the Brazilian context, where there is difficulty in providing support in primary care, with limited coverage of Primary Health Units (UBS) and Family Health Units (USF) for clarification of residual doubts that arise after returning home and assistance in the face of possible complications [11,12]. Preventable adverse events directly impact the quality and continuity of care and, consequently, early (re)hospitalizations [6,13]. Training throughout hospitalization and follow-up after discharge raise awareness and engagement, which are important properties that help in the acquisition of mastery [14,15].

Mastery is an important result indicator, characterized by the mastery of new skills and the ability to manage situations in the new context of intense transformations. Mastery is supported by self-confidence, the ability to make decisions, and knowledge and cognitive ability. It also depends on personal characteristics, formal and informal supports, situational awareness and engagement to adapt. These elements influence a smooth transition from hospital to home, providing continuity of care [16].

Fundamental points must be identified so that they can be addressed and enhanced in health education during hospitalization, while also considering the relevance of ongoing education for the care team, whose view must go beyond hospitalization, valuing life contexts for understanding and adequate training based on individual needs.

Therefore, the objective of the study was to understand aspects involved in the acquisition of mastery, by home caregivers, of handling gastrostomy care after an educational intervention during hospitalization and telemonitoring upon the return home.

## 2. Materials and Methods

### 2.1. Design and Setting

This is a descriptive and qualitative study whose interest was to understand the aspects related to the phenomenon of “acquisition of mastery by caregivers in continued care with gastrostomy, after educational practices during hospitalization and telemonitoring upon returning home”.

The scope of mastery was considered to be the development of skills to provide routine care, the interpretation of basic signs and symptoms of complications and skin injuries in contact with the device, the development of skills and abilities for decision-making in the face of complications, and the need to request help from members of the monitoring team and/or health services to search for information and resolve doubts.

The situations reported and discussed here refer to follow-ups between November 2022 and July 2023 in the neuromusculoskeletal unit of a large public university hospital, a reference in medium and high complexity care, whose services occur exclusively through the Brazilian Public Health System (SUS) [17].

### 2.2. Population

The researchers selected the sample from a university research and extension project that monitors high-dependency people, according to the Fugulin scale classification [18], and their family members/caregivers during hospitalization and after discharge through telemonitoring, considering caregivers who met the following inclusion criteria: (1) caregivers of adults or older people with gastrostomy who were discharged from the hospital to their home; (2) those who were present, during hospitalization, for health education carried out by the project team; and (3) those who did not have continuous formal support for carrying out PEG care. As a result, 15 caregivers met the inclusion criteria for this research.

### 2.3. Information about the Fugulin Scale and Academic Project

The Fugulin scale, developed and validated by a Brazilian nurse, is a tool used in the context of Brazilian health services to assess the level of nursing care required by hospitalized patients. The scale evaluates various aspects of patient dependence, including mental status, oxygenation, vital signs, motility, ambulation, feeding, body care, elimination, and therapy [18].

The main project aimed to identify the individual and specific needs of highly dependent individuals (adults and older people), as well as the needs of their family members and other caregivers. The objective was to plan the transfer of care to the patient’s home, with subsequent monitoring for three months after discharge. The monitoring plan involved weekly calls in the first month after discharge, fortnightly calls in the second month, and a single call in the third month, to assess the patient’s adaptation and the acquisition of care mastery. All people who agreed to participate in the project signed a consent form, and their data were organized in an Excel spreadsheet.

### 2.4. Educational Intervention

The educational intervention focused on providing health education to family caregivers of people using a PEG tube (or who would undergo a surgical procedure for its insertion) due to dysphagia related to an underlying neurological disease. These individuals were classified as having high dependence and low autonomy in care [18,19].

The educational activities took place in the ward on a previously agreed day and time, lasting approximately 40 min, according to the caregivers’ demands and skills, before or after tube installation, depending on the availability of caregivers and the team responsible for providing guidance. The caregivers’ time in relation to receiving the indication of the device and elaborating the situation was also respected.

The educational intervention planning and execution took place in four stages:

#### 2.4.1. Stage 1—Instructing Caregivers about the Device

When a person was eligible for the intervention, the first contact was made by the matrix project team. The study was then presented to the caregivers, and if they accepted the invitation, their sociodemographic data were collected, organized, and stored in Excel spreadsheets.

Patients could be included in the matrix project upon admission or during hospitalization if their degree of dependence reached the inclusion criterion score of 21 to 26 points on the Fugulin scale [18,19]. At the study unit, the indication for the placement of a PEG tube occurred after evaluation by the care team, particularly speech therapy professionals, after rehabilitation attempts.

After identifying the need for the PEG procedure and scheduling the placement, the research team planned the best time for the first orientation meeting with the family. During this initial meeting, the care team’s previous guidance regarding the indications, instructions related to the tube, and the importance and necessity of its use were reinforced and reviewed. Any doubts that arose were addressed, and emotional support was provided regarding the anxieties and insecurities presented by the caregivers. Finally, the best day and time for the educational intervention on the use of the PEG was scheduled.

#### 2.4.2. Stage 2—Training Caregivers to Handle the Tube

For the educational intervention, the number of meetings scheduled depended on the participant’s demand. This phase aimed to provide an understanding of care regarding cleaning techniques; administration of diet, liquids and medications; as well as guidance on fixing the tube; managing complications (leaks, signs of infections, loss of the tube, obstructions); and organizing material for care. Additionally, guidelines were provided related to oral hygiene care, skin care, and the correct positioning of the patient in the bed at home, which is essential for the care of bedridden people with dysphagia and functional dependence.

**Figure 1:**
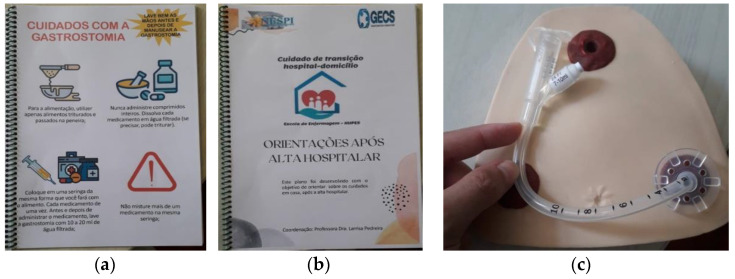
Resources used for educational activities with caregivers Scheme 2024. (**a**) a booklet about gastrostomy care. (**b**) a booklet of “Guidelines after hospital discharge”. (**c**) “anatomical specimen of abdominal region with gastrostomy tube used during practical training”.

To create a realistic simulation and promote better understanding, the researchers used an anatomical piece of the abdominal region with an attached PEG tube, as well as a 60 mL syringe, gauze, and drinking water. Scientifically supported booklets created by the main research team (Figure 1) were also used to reinforce and illustrate the guidelines, and were later sent via messaging application for observation, reading, and discussion of any doubts.

#### 2.4.3. Stage 3—Feedback Provided during and after Training

During the training sessions, feedback was provided to the caregivers regarding the skills acquired, encouraging them and identifying points to improve the guidance. Specific information was highlighted, such as the consistency of the food to avoid tube obstruction, the ideal temperature to administer the diet, the rate at which the diet should be administered, ways to clean before and after administering food and medications, and the identification and recognition of complications related to the tube and skin.

After the training, the caregivers were invited to view the tube on their family member, as the tube model they would be using at home was often different from the one used during the training. Immediately after the gastrostomy procedure was performed, the telemonitoring follow-up method began, which would continue for three months, with the first call occurring in the first week after discharge.

#### 2.4.4. Stage 4—Follow-Up after Discharge to Observe the Outcome of Interest

The follow-up after discharge was conducted through telemonitoring, which involved seven calls over a three-month period and did not prevent the caregivers from contacting the project team at other times, in case of doubts, either by phone calls or via messaging application. During each follow-up call, a form was filled out with the results, and the caregivers were able to raise any doubts or questions, which were then addressed and clarified by the project team.

### 2.5. Data Collection and Analysis

To analyze the outcome of interest, forms from the matrix project were used to collect sociodemographic data: sex, age, education, profile (formal or family), time providing care, awareness of the health status of the person under care, formal support on a specific or informal basis, confidence about continuity of care, number of calls received during telemonitoring, and use of an application to exchange messages. During the telemonitoring after discharge, additional information was collected using the matrix project monitoring form, such as the use of a support network (informal or formal), doubts about home care and prescriptions from health professionals, and other demands.

All of the data were organized in an Excel spreadsheet for analysis and discussion of the content based on the Transitions Theory, a mid-range nursing theory proposed by the Egyptian-American Nurse Dr. Afaf Ibrahim Meleis [16]. The information in the text was described according to the Consolidated Criteria for Reporting Qualitative Research (COREQ), translated and validated version for the Portuguese language [20].

### 2.6. Ethical Considerations

The matrix project was approved by the Ethics Committee of the University Hospital Professor Edgard Santos at the Federal University of Bahia under opinion 5.282.090. This approval was in compliance with resolutions no. 466/2012 [21] and no. 510/2016 [22], which determine specific guidelines and ethics for human and social sciences research.

## 3. Results

Between November 2022 and July 2023, the research team monitored 52 people with high dependency on care admitted to the neuromusculoskeletal ward. Of these, 17 underwent gastrostomy due to severe dysphagia after neurological impairment from a stroke, and 15 caregivers met the inclusion criteria for this section, as exemplified in Table 1.

All patients who needed a PEG tube were bedridden and dependent on care for Basic Activities of Daily Living (BADL) and Instrumental Activities of Daily Living (IADL). The educated caregivers had no prior knowledge about the PEG tube, but they all had a basic education and a good understanding of learning.

In the first stage of instruction, after the indication for gastrostomy, it was noticed that the health condition and recommended therapy had an impact on the caregivers, most of whom were family members. They were scared and afraid of managing the PEG, mainly in continuing care safely. The most frequent doubts were related to the indication of PEG instead of the use of the nasoenteral tube, its possible repercussions in the face of frailty, and the possibility of the patient returning to the functional capacity of swallowing and eating after the PEG procedure.

In the second stage, the caregivers were invited to training, which was conducted in an external area to avoid distractions. It was noticed that the participants were aware and engaged in caring for and safely managing the device at home, as they wanted to be prepared for possible complications and act on them. The doubts reported were related to a better understanding of tube care, and the need to share knowledge with other people in the family on whom they counted for support.

In the third stage, the feedback enhanced the caregivers’ engagement for continuity of care. When the caregivers realized that they were making progress, they demonstrated satisfaction with the training, greater confidence, and less fear related to possible adverse events. These reactions were evident in the more fluid and elaborate reports, questions consistent with their social and economic realities, and less resistance to learning when compared to the initial approach.

In the fourth stage, through telemonitoring, it was noticed that the first month after hospital discharge was the most critical period for the caregivers, especially the first and second weeks, considering that these people were getting adapted to family dynamics for shared care, along with work. Some caregivers (C2, C3, C4, and C6)—Table 1 had to give up their work activities and dedicate themselves to this care on top of their other usual responsibilities.

In these cases, despite instructions throughout hospitalization and follow-up, complications arose related to the formation of granulomas in the PEG insertion stoma of seven people receiving care (C2, C3, C4, C5, C8, C11, and C12)—Table 1, tube obstruction in three (C2, C3, and C14), and accidental loss of the tube in two (C6 and C7)—Table 1, which occurred twice in the same person (C6)—Table 1. Regarding these incidents, there was real-time contact with the telemonitoring team, who, in stages, redid the guidelines provided at the hospital, resolving the problem and referring patients to health units, as shown in Table 2.

It should be noted that caregivers were aware of their difficulties and limits related to care. Through support from the matrix project team, hospital professionals, and other professionals on a specific basis (Primary Health Care and Home Care), they managed to develop the necessary skills for care and management, especially in the face of complications, assertively seeking out members of the research team and/or health services. The exceptions were C7 and C13 (Table 1), who, despite not having formal support, chose not to continue with telemonitoring, thus resulting, for example, in pneumonia, due to bronchoaspiration of food, and death two months after C7’s family member was discharged from hospital.

## 4. Discussion

The first stage of the training process revealed the emotional fragility of the caregivers, resulting from fear and uncertainty. This factor was also evidenced in a study of caregivers of older people undergoing PEG in Taiwan, where the risks and benefits of this feeding route were not specified by health professionals, leaving caregivers worried, confused, and insecure about individually consenting to the procedure, resulting in prolongation of hospitalization time and greater exposure to adverse events related to nasoenteric tube use [23].

In the present study, the caregivers had information about the need for gastrostomy, although the communication with the unit’s care team was insufficient in some cases (C2, C3, C4, C8, and C14—Table 1), requiring further explanations from the research care team. Insufficient communication to resolve caregivers’ doubts can negatively impact the acquisition of necessary skills and mastery, which are important outcome indicators for a healthy hospital-to-home transition [16].

This finding is supported by a study that identified factors associated with the cognitive, emotional, psychomotor, and relational skills of caregivers in home care, which emphasized that efficient communication between health professionals and caregivers was essential for them to continue with home care in a planned and safe manner [24]. Intercommunication contributed to the acquisition of new skills, obtaining relevant information about caring, and, consequently, acquiring mastery [24].

Combined with insufficient communication, the emotional and physical exhaustion resulting from the caregivers’ stay in the hospital can have a negative impact on the understanding of information. In the context of the participants in this study, the majority (C4, C5, C6, C8, C9, C12, C13, C14, and C15—Table 1) were from municipalities located in the inland areas of the state, and with a weak informal support network (C8, C12, C14, and C15—Table 1) to share monitoring throughout hospitalization. This situation led to exhaustion, with physical and mental fatigue in caregivers, who also demonstrated a feeling of insecurity regarding the continuity of care, demanding, after discharge, in addition to conventional calls, interactions with text message via app to promote emotional support and ensure greater safety, and resolve residual doubts related to tube care and management of signs and symptoms indicative of complications.

Discharge planning specifically for the continuity of care with PEG for bedridden people with high care dependency should be carried out throughout the hospitalization and continued after returning home. This is because it is during hospitalization that there is an opportunity to get closer to the people involved, knowing their context for planning aligned to their needs. It is crucial to establish transitional care that focuses on the individual and their family, as this is important when a person is discharged from the hospital and returns home. At this point, the person takes on care responsibilities that were previously handled by healthcare professionals, and these responsibilities should now be taken on by the support network [25,26]. However, the support network is often confused and insecure about medication reconciliation, use of devices, and management of signs and symptoms.

The interaction among affected people, family caregivers, and the healthcare team strengthens process indicators in the transition of care. These indicators include understanding the affected person’s health condition and the need for continuity of care, connecting with the healthcare team, considering guidelines and strategies as a form of support, and interacting and inter-relating through the exchange of experiences. These indicators are important for developing confidence and coping strategies in the new context [16]. This interaction also contributes to a collaborative relationship in teaching care, in this case, PEG. A study effectively demonstrated the significance of interaction between nurses and young adults during the care transition process, observing that when healthcare professionals prioritize operational tasks over building relationships with patients, the latter feel insecure, objectified, and helpless, leading to distrust, low adherence, and abandonment of care plans [27].

In this study, nine caregivers (C1, C2, C3, C4, C6, C7, C9, C14, and C15—Table 1) received training before the insertion of the PEG tube into their family members. This training took place on the hospital’s external premises, in a calm environment, without the movement of professionals or other interference, using accessible language and no technical terms. This organization contributed to ensuring that there were no interruptions and distractions, resulting in better learning at the end of the health education.

This finding is supported by a study that explored the experiences of gastrostomy insertion from the perspective of affected people and informal caregivers, which found that participants did not absorb the information provided and/or were unprepared for the practical aspects related to food administration [28]. This was attributed to the lack of guidance before creating the gastrostomy (pre-surgical period) and the inadequate training environment, as they were trained within a neurological ward that was busy and had distractions [28].

Another investigation with caregivers of people undergoing PEG in a university hospital in Turkey observed that the content of the training provided during hospitalization was insufficient and short, making information comprehension difficult [6]. Caregivers were afraid and stressed during the training, as they were not confident that they would be able to reproduce the care, and they pointed out that training should not only occur once and verbally, but rather with practical and demonstrative experiences [6].

In this study, the training did not have a defined end time and varied depending on the caregiver’s acquisition of skills. Furthermore, the realistic simulation with an anatomical piece promoted an interactive and participatory space between caregivers and the team carrying out the educational intervention. This is a practical, repetitive teaching method that influences the ability to understand, engage in the search for information, and clarify residual doubts [29], allowing the development of mastery and prevention of complications related to inadequate care.

During the intervention, caregivers had space to share doubts and concerns, and from there, received clarifications. Subsequently, they were invited to train and demonstrate learning through speech and practice on the anatomical piece under supervision, receiving feedback on their skill and knowledge acquisition, thus driving the decision as to when training could be completed. Studies have shown that feedback is an essential strategy in the process of evaluating learning and skill acquisition [30]. When positive, it can motivate and involve caregivers; however, when negative, it works as a starting point for reorganizing the educational action, with new attempts to put the desired skill into practice [30].

After the family member was discharged, monitoring continued via telemonitoring through telephone calls, audio and/or videos via messaging app, aiming to ensure good adaptation at home. In the cases reported here, this monitoring resulted in a better understanding of the need to use the tube, its safe and appropriate handling, safety in managing signs and symptoms, cognitive ability in the face of complications, clarification of doubts, qualified listening, and emotional support. Furthermore, monitoring promoted a feeling of security in caregivers and allowed for better engagement perceived by feedback on messages and guidance.

The positive impact of this care was observed, as all caregivers demonstrated insecurity and anguish regarding discharge before the intervention. A study revealed that telemonitoring favored the maintenance of bonds and adherence to treatment; supported self-care; and provided confidence and support for hospitalized people and their socio-family network [31]. This technology has the potential for engagement, making the affected person and their caregivers protagonists of the therapeutic process [31].

The first month after discharge, specifically the first week, was a delicate period of intense transformations and new meanings for caregivers, due to fear and apprehension of what could happen, of not being able to manage care, which included tube management. Furthermore, these people were still faced with the need to align this reality with work and restructure family dynamics to share assistance. This fact was particularly noticeable in those who had played the role of caregiver for less than 6 months (C2, C3, C4, C6, C7, C9, C11, C12, C14, and C15—Table 1), who made an average of 7.6 phone calls, that is, 3.6 more than those who had been providing care for more than 12 months (C1, C5, C10, and C13—Table 1, possibly because they lacked experience and perceived, in telemonitoring, the opportunity to acquire knowledge and provide safer care.

Regarding the aspect of having telephone contact in the first week after discharge, the authors discuss the importance of this approach to understand how the hospital–home transition occurred, and whether there were doubts related to care. If the caregiver seems insecure for any reason, professionals must assess their needs and act on them [32].

In this study, all caregivers experienced the transition in the health–illness process, and in addition, seven (C2, C3, C5, C6, C7, C14, and C15—Table 1) experienced situational transitions. Transitions in the health–disease process resulted from the transition from hospital to home, while situational transitions occurred due to the addition or subtraction of people in the environment and/or redefinition of roles in the family context [16].

The situational transitions contributed to four caregivers (C2, C3, C4, and C6—Table 1) giving up their work, leisure and self-care activities, resulting in emotional fragility and musculoskeletal pain related to this role. These setbacks can interfere with engagement and interaction with professionals to discuss doubts, due to the lack of time resulting from exclusive and routine care. Moreover, ergonomic diseases and repetitive strain injuries are situations that can occur in people who provide intense and continuous care for bedridden and dependent individuals [33], interfering with the time and quality of care provided.

Regarding the occurrence of accidental loss of the tube, telemonitoring, when activated, provided support by giving guidelines related to cleaning the ostium with warm water, protecting with gauze and immediate transfer to the local hospital, according to recommendations, as the municipality in which the patient was located did not have Emergency Services activated [34]. On both occasions of this incident, there was rapid and timely response to the displacement, considering that the family member was aware that the PEG orifice could occlude according to the guidelines provided at the hospital. The problem related to the gastrostomy tube obstruction was also resolved when telemonitoring was activated, with instructions through video call via the application. In both situations, guidance regarding complications occurred in real time, with resolution of the problem.

Regarding granuloma care, the guidance was to use 20% sodium chloride (NaCl) due to its ease of access in Primary Health Units and/or lower cost in local drugstores. The practice consisted of moistening gauze with 20% NaCl and applying it around the lesion for a period of five to ten minutes, with it behaving like a hypertonic dressing. The hypertonic solution causes an osmotic imbalance, in which the cells lose fluids to the more solute-concentrated medium (gauze with 20% NaCl), reducing the hypergranulation tissue [35]. The therapeutic effects were satisfactory after guidance and monitoring.

In all cases presented, the caregivers were aware of the family member’s health status. This awareness is characterized when that event gains meaning, promoting engagement to address the situation. This was realized through the sending of photos and videos of caregivers manipulating the tube, real-time video calls to answer questions and/or share progress in rehabilitation and recovery, as well as the search for additional information provided by the UBS team as per observed in the C2, C3, C4, C7, and C14 (Table 1) situations.

A study involving 21 caregivers in Turkey observed that a lack of awareness interfered with the acquisition of mastery, leading these people to make unsafe and hasty decisions in the face of incidents [6]. As an example, they unblocked the PEG with pieces of wire and injected saline solution into the tube [6]. In cases of leakage, a situation that may occasionally occur, they fixed plaster to the parts of the tube that drained liquids or cut these parts and reinserted the tube [6]. In view of the above, we can see the importance of discharge management promoted by the multidisciplinary team and follow-up after return home, to ensure an effective hospital–home transition, adaptation, and continuity of care.

This study is valuable for nursing professionals as it encourages reflection on clinical practice in the hospital environment, the barriers that need to be overcome, and how actions for discharge planning impact the awareness and engagement of caregivers in the home environment. It highlights the need for follow-up to clarify any remaining doubts and preventive practices, resulting in person- and family-centered care, maintenance of autonomy, continuity of care at home, reduction of unfavorable health outcomes, early readmissions, and a decrease in hospital costs.

This study has limitations due to the specific target population and the fact that its findings may not be widely applicable. While this study did not face this issue, it is important to note that not all patients and caregivers have access to the internet or suitable mobile devices for remote monitoring. Therefore, nurses should use hospital time to plan a safe and effective discharge and provide training and health education. The success of telemonitoring depends, in part, on the skills acquired in the hospital. Additionally, to improve monitoring outcomes, it is important to customize a call plan based on the patient’s profile, considering their pre-existing conditions and the skills of the caregivers.

## 5. Conclusions

The process for home caregivers to acquire mastery in gastrostomy management involves a series of aspects that can influence whether or not the transition is safe. These include the use of appropriate spaces for training; the provision of personalized time for each caregiver; a combination of theoretical and practical demonstrations with constant feedback; transparent, welcoming, and accessible communication; and continuous monitoring of adaptation, especially during the first month after hospital discharge, since complications arose despite the educational intervention. Still, the caregivers demonstrated mastery in managing them.

In addition, it was observed that the physical and emotional exhaustion caused by the lack of knowledge about the device, the experience of accompanying the family member in the hospital, and a weak support network influenced the acquisition of mastery through the greater insecurity of these people regarding the continuity of care. The specific details of the procedure, such as its restrictions and possible complications, are also factors that should be considered, as they can promote insecurity in caregivers.

To ensure a safe transition in situations involving people using a gastrostomy device, the multidisciplinary team must begin discharge planning from the beginning of the hospitalization, setting aside enough time to explain the procedure and its purpose and to carry out simulated training adjusted to the reality of each caregiver, strengthening them with educational material. In this scenario, nursing plays a leading role, given its proximity, frequency of contact, and constant communication with patients and their caregivers.

## Figures and Tables

**Table 1:** Characterization of caregivers participating in the educational intervention. Salvador, Bahia, Brazil, 2023.

Code Name	Sex/ Age	Level of Education/ Occupation/ Location and Area of Residence	Caregiver Profile/ Time Spent Caring	Aware of the Situation	Formal Support on a Timely Basis/ Informal Support	Safety for Hospital Discharge/ Telemonitoring Calls	Use of Messaging App for Residual Doubts
C1	F/ 42	Unfinished elementary school/ no defined occupation/ inland city/urban	Formal/ >12 months	Yes	No Yes	Insecure/ 5 calls	Yes
C2	F/ 67	Finished undergraduate degree/ social worker/ capital/urban	Family member/ <6 months	Yes	Yes Yes	Insecure/ 12 calls	Yes
C3	F/ 58	Finished undergraduate degree/ pharmacist/capital/urban	Family member/ <6 months	Yes	Yes Yes	Insecure/ 9 calls	Yes
C4	F/ 57	Finished elementary school/ housewife/ inland city/rural	family member/ <6 months	Yes	No Yes	Insecure/ 7 calls	Yes
C5	F/ 57	Finished high school/ hairdresser/ inland city/urban	Family member/ >12 months	Yes	No Yes	Insecure/ 4 calls	Yes
C6	F/ 30	sales consultant/ inland city/urban	Family member/ <6 months	Yes	No Yes	Insecure/ 8 calls	Yes
C7	M/ 52	Unfinished elementary school/ mason/ capital/urban	Family member/ <6 months	Yes	No Yes	Insecure/ 3 calls	No
C8	F/ 65	Unfinished elementary school/ housewife/ inland city/urban	Family member/ 6 to 12 months	Yes	No Yes	Insecure/ 7 calls	Yes
C09	F/ 43	Unfinished elementary school/ housewife/ inland city/rural	Family member/ <6 months	Yes	No Yes	Insecure/ 6 calls	Yes
C10	F/ 20	Finished high school/ e-commerce businesswoman/ capital/urban	Family member/ >12 months	Yes	No Yes	Insecure/ 5 calls	Yes
C11	M/ 39	Finished undergraduate degree/ pharmacy assistant/ inland city/rural	Family member/ <6 months	Yes	Yes Yes	Insecure/ 4 calls	Yes
C12	M/ 45	Unfinished elementary school/ driver/ inland city/ rural	Family member/ <6 months	Yes	No Yes	Insecure/ 7 calls	Yes
C13	F/ 52	Finished high school/ housewife/ inland city/urban	Family member/ >12 months	Yes	No Yes	Secure/ 2 calls	No
C14	F/ 43	Finished elementary school/ housewife/ inland city/urban	Family member/ <6 months	Yes	No Yes	Insecure/ 12 calls	Yes
C15	F/ 25	Unfinished higher education/ gastronomy student/ inland city/rural	Family member/ <6 months	Yes	Yes Yes	Insecure/ 8 calls	Yes

**Table 2:** Characterization of patient complications, effectiveness of training and remote monitoring actions, and recorded outcomes. Salvador, Bahia, Brazil, 2023.

Code Name	Complications of the Patient	Effectiveness of Training and Telemonitoring Interventions	Results Observed
C2; C3; C4; C5; C8; C11; C12	Granuloma in the PEG insertion stoma	- It was recommended to clean the PEG ostium with neutral soap, and after bathing, moisten a gauze with 20% sodium chloride (NaCL), apply to the granuloma and leave it in place for 5 to 10 min.	- Granuloma remission in 6 to 8 days.
C2; C3; C14	PEG obstruction	- The caregivers understood that they should not ingest acidic solutions or insert objects to try to unblock the obstruction. Instead, they contacted the research team. - On all three occasions, the administration of warm water was advised using a 5 mL syringe, as the greater pressure exerted would help clear the obstruction.	- Immediate unblocking. - Caregivers were instructed on the consistency of food and the importance of washing the PEG tube before and after administration to avoid new obstructions.
C6; C7	Accidental loss of tube	- On both occasions when the feeding tube was accidentally dislodged, the caregiver (C6) cleaned the opening with mild soap, covered it with gauze, and promptly took the patient to the emergency care unit. She was aware that the PEG opening could become blocked within a few hours, as per the instructions she had received. When the incident occurred, the caregiver contacted the project team to report the actions being taken, without requiring any further information.	- Quick and timely transfer to the emergency care unit. - Regulation for the reference service for new PEG reinsertion.
		- After the incident, caregiver C7 immediately contacted the project team. Due to fear and nervousness, he was unsure about what to do. However, he was advised to clean the opening with neutral soap, cover it with gauze, and seek medical attention at the nearest health service. - The project members contacted the local hospital where (C7) was admitted to report the patient’s health history, the time since discharge after insertion of the PEG, and recommended referral to the referral hospital for reinsertion.	- Quick and timely transfer to the emergency care unit. - Regulation for the reference service for new PEG reinsertion.

## Data Availability

The datasets generated and analyzed during the current study are available from the corresponding author. They can also be shared with the journal for review upon request.
